# Optimized production and safety evaluation of hispidin‐enriched *Sanghuangporus sanghuang* mycelia

**DOI:** 10.1002/fsn3.1469

**Published:** 2020-03-03

**Authors:** I‐Chen Li, Chang Cheng Chen, Sen‐Je Sheu, I‐Hsuan Huang, Chin‐Chu Chen

**Affiliations:** ^1^ Biotech Research Institute Grape King Bio Ltd Taoyuan Taiwan; ^2^ Department of Food Science and Biotechnology Da‐Yeh University Changhua Taiwan; ^3^ Institute of Food Science and Technology National Taiwan University Taipei Taiwan; ^4^ Department of Bioscience Technology Chung Yuan Christian University Taoyuan Taiwan; ^5^ Department of Food Science, Nutrition, and Nutraceutical Biotechnology Shih Chien University Taipei Taiwan

**Keywords:** hispidin, *Phellinus linteus*, safety assessment, *Sanghuangporus sanghuang*, toxicity

## Abstract

*Phellinus linteus*, also known as the sanghuang mushroom, is a medicinal mushroom that has been recognized as beneficial to health for more thousands of years. Among its diverse valuable secondary metabolites, the yellow‐brown styrylpyrone pigment hispidin has garnered significant attention due to its various pharmacological effects. However, recently after detailed morphological and molecular phylogenetic studies, the correct scientific name of the true sanghuang strains was shown not to be *P. linteus* but *Sanghuangporus sanghuang*. As the incorrect binomial name *P. linteus* has long been misleadingly referred, there is a need to evaluate the safety of *S. sanghuang*. Moreover, the growing conditions can impact the secondary metabolite profile of the fungi. Hence, this study is the first to optimize hispidin production and to investigate the genotoxic and oral toxic effects of hispidin‐enriched *S. sanghuang* mycelia. In order to induce the biosynthesis of hispidin, 15 different culture media consisting of five carbon sources, five nitrogen sources, and five initial pH conditions were screened. Glucose and yeast extract at an initial pH of 5 were found to be the most suitable carbon and nitrogen sources, respectively, for the optimal growth and production of hispidin. Moreover, the production of hispidin was 3 mg/g in a 20‐ton bioreactor under optimal conditions. Furthermore, the ames test, in vitro chromosome aberration test, acute oral toxicity test, and bone marrow micronucleus test were used to detect toxicological properties of 3 mg/g hispidin‐enriched *S. sanghuang* mycelia. In all tests, there was no statistically significant difference between the mycelia and the negative control. Based on the results obtained, the present study demonstrates that 3 mg/g hispidin‐enriched *S. sanghuang* mycelia has a very low order of toxicity, which supports its safety for human consumption.

## Graphical abstract

The results of the present study concluded that glucose and yeast extract at an initial pH of 5 are the optimal conditions for the production of hispidin by *Sanghuangporus sanghuang* mycelia. Moreover, the absence of any remarkable adverse effects in the Ames test, in vitro chromosome aberration test, acute oral toxicity test, and bone marrow micronucleus test demonstrated that 3 mg/g hispidin‐enriched *S. sanghuang* mycelia is safe for human consumption.

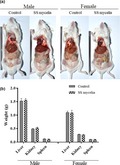

## 1 INTRODUCTION

For thousands of years, numerous mushrooms and their ingredients have been valued in traditional oriental therapies (Elsayed, El Enshasy, Wadaan, & Aziz, 2014). *Phellinus linteus*, also known as “sanghuang” in China, “meshimakobu” in Japan, and “sangwhang” in Korea, is a well‐known fungus and has been recognized as beneficial to health for more than 2,000 years (Chen, Tian, Miao, & Zhao, 2016). Numerous studies have demonstrated that *P. linteus* possesses antitumor, anti‐inflammatory, anti‐allergic, anti‐angiogenic, antioxidant, hypoglycemic, hypolipidemic, and immunomodulatory activities (Liu, Wang, Li, Mei, & Liang, 2019; Sliva, 2010). The pharmacological properties of *P. linteus* are often associated with the presence of high‑molecular‑weight and low‑molecular‑weight compounds. For example, hispidin, a polyphenol compound particularly derived from the genus *Phellinus* species, has garnered significant attention due to its antioxidant (Park et al., 2004), anti‐obesity (Tu & Tawata, 2014), anticancer (Lim, Lee, Park, Kim, & Lim, 2014), antiviral (Awadh Ali, Mothana, Lesnau, Pilgrim, & Lindequist, 2003), anti‐allergic (Tamrakar, Fukami, Parajuli, & Shimizu, 2018), antibacterial, and anti‐inflammatory effects (Shao, Jeong, Kim, & Lee, 2015). Considering *P. linteus* holds vast sources of biologically active constituents, nutritional/dietary supplements containing *P. linteus* are on the rise in Western countries (Sliva, 2010).

However, although consuming mushroom products is thought to provide medicinal benefits, safety concerns have frequently been raised due to its taxonomy and the uncertainty surrounding secondary fungal metabolite productions. For example, a recent study has revealed that out of 37 fungal natural product articles every year, 31% provided fungal identification based solely on its morphology (Raja, Miller, Pearce, & Oberlies, 2017). Although phenotypic characters are the foundation of fungal taxonomy, such method can be objective due to the variations in morphological traits. Additionally, the taxonomy of *P. linteus* has been difficult because of the inadequacy of morphological distinctions within closely allied species (Han et al., 2016). To overcome such drawback, analysis with nucleotide sequences has been developed into becoming the new gold standard for differentiating these species (Raja et al., 2017). After detailed morphological (Dai, 2010) and molecular phylogenetic studies (Wu et al., 2012), *P. linteus* was shown not to be the correct name, but as *Sanghuangporus sanghuang* instead (Sheng H. Wu, T. Hatt. and Y.C. Dai) (Han et al., 2018).

As the incorrect binomial name *P. linteus* has long been misleadingly referred, there is a need to evaluate the safety of true sanghuang strains. Moreover, the active substance within sanghuang strains can act in beneficial or harmful ways as it will occasionally have beneficial properties at low concentrations but could be toxic at high concentrations (Sorell, 2015). To date, *S. sanghuang* has not been evaluated for its safe human consumption using modern techniques. Hence, the present study is designed to evaluate the health risk assessment of 3 mg/g hispidin‐enriched *S. sanghuang* (GKSS) to determine its safety for use via in vitro and in vivo toxicological testings.

## 2 MATERIALS AND METHODS

### 2.1 Authentication of *S. sanghuang* strain

The study was conducted in accordance with the Basic & Clinical Pharmacology & Toxicology policy for experimental and clinical studies (Tveden‐Nyborg, Bergmann, & Lykkesfeldt, 2018). The *S. sanghuang* mycelia (GKSS) used in this study were isolated from the fruit body harvested from the mountainous region in Guanxi Township (Hsinchu, Taiwan) and deposited at the nearby herbaria after strain authentication. Genotypic identification of the *S. sanghuang* GKSS strain was authenticated by the fungal ITS sequences according to the previous study (Wu et al., 2012).

### 2.2 Optimization of hispidin production of *S. sanghuang* mycelia

To select a suitable medium for the production of hispidin, five carbon sources, five nitrogen sources, and five initial pH conditions were tested in flasks at volume of 250 ml. Glucose, sucrose, lactose, maltose, and starch were used as carbon sources, where each was added into a basal medium (with MgSO_4_) at a concentration of 1 g/L. For nitrogen sources, yeast extract, peptone, whey peptone, malt extract, and gluten were utilized, where each was added into a basal medium at a concentration of 0.3 g/L. To determine the ideal initial pH for hispidin production of *S. sanghuang* mycelia, the medium adjusted to pH 4, 5, 6, 7, and 8 were adopted. After 10 days of incubation with different parameters, the quantity of hispidin was evaluated.

### 2.3 Mass production of hispidin‐enriched *S. sanghuang* mycelia

A mycelia agar block (1 cm^3^) was transferred to a 1.0 L optimized broth (composed of 1% glucose, 0.3% yeast extract, and 0.05% MgSO_4_, adjusted to pH 5) and cultivated at 25°C for 1 week on a rotary shaker at a speed of 120 rpm. For mass production, this fermentation process was scaled up from a shake flask to a 500‐L and a 20‐ton fermenter for 10 days. The collected hispidin‐enriched *S. sanghuang* mycelia were then harvested, lyophilized, grounded into powder, and stored in a desiccator at room temperature for further analysis with high‐performance liquid chromatography (HPLC).

### 2.4 Hispidin analysis

Hispidin analysis was performed according to a previously reported method with slight modifications (Jang et al., 2010). In brief, 20 g powder was placed into 1,000 ml of absolute alcohol, filtered through Whatman filter paper No. 4, and concentrated through a rotary evaporator (R‐220, Büchi Labortechnik AG) to obtain dried ethanolic extract. The qualitative determination of hispidin in *S. sanghuang* mycelia ethanolic extract was carried out by a HPLC equipment (CM‐5000 series, Hitachi) connected to a Chromaster 5430 diode array detector (DAD). Hispidin was separated on a Kinetex column (4.6 × 150 mm, 5 μm), using a linear gradient of methanol and 0.1% formic acid, from 30:70 to 100:0 in 13 min followed by 30:70 in 2 min. The column temperature was set at 40°C, the flow rate was set at 1.0 ml/min, and chromatograms were processed at 370 nm.

### 2.5 Bacterial reverse mutation test (Ames test)

The Ames test was performed in accordance with a previous study (Maron & Ames, 1983) and guidance of Organization for Economic Cooperation and Development (OECD) Guideline for the testing of chemicals #471: Bacterial reverse mutation test (OECD, 1997a). Dimethyl sulfoxide was used as the negative control while 4‐nitroquinoline‐N‐oxide, sodium azide, mitomycin C, 9‐aminoacridine, 2‐aminoanthracene, benzo [a] pyrene, and 2‐aminofluorene (Sigma‐Aldrich) were used as positive controls. In brief, 100 μl of *Salmonella typhimurium* strains TA98, TA100, TA102, TA1535, and TA1537 (MolTox Inc.) was exposed to hispidin‐enriched *P. linteus* mycelia at the highest dose of 5,000 µg/plate and four doses of 313, 625, 1,250, and 2,500 µg/plate as the dose range in the presence and absence of metabolic activation. The mixtures were added to 2 ml of molten top agar containing 0.5 mM of histidine/biotin, poured on the surface of minimal glucose agar plates, and then incubated for 48 hr at 37°C prior to revertant colonies counting. Each concentration of the test substance was set up in triplicates. A positive result is considered when there is a dose‐dependent increase of revertant colonies in at least one of the tester strains without cytotoxicity and a twofold increase of spontaneous revertants over the negative control.

### 2.6 In vitro chromosome aberration test

The in vitro chromosome aberration test was conducted in accordance with the OECD Guideline for the testing of chemicals #473: In vitro mammalian chromosome aberration test (OECD, 1997b). To determine the highest concentration of test substance to be used in this study, hispidin‐enriched *S. sanghuang* mycelia at dose levels of 0, 1.25, 2.5, and 5 mg/ml were first exposed to CHO‐K1 cells (BCRC 60006) in the presence and absence of a metabolic activation system. Based on the dose‐range finding results, 5 mg/ml of hispidin‐enriched *S. sanghuang* mycelia was decided as the maximum dose since CHO‐K1 cells exhibited <50% of cytotoxicity after 3 and 20 hr treatment in the absence of S9 activation, and 1.25 mg/ml of hispidin‐enriched *S. sanghuang* mycelia was decided as the maximum dose since it induced approximately 50% of cytotoxicity after 3 hr treatment in the presence of S9 fraction. All experiments were performed in duplicate. In brief, CHO‐K1 cells were exposed to hispidin‐enriched *S. sanghuang* mycelia for 3 hr with and without metabolic activation and for 20 hr in the absence of metabolic activation. After treatment, 0.1 μg/ml demecolcine solution (Gibco) was added to each culture approximately 20 hr after the initial treatment and further incubated for 4 hr prior to harvesting. Cells were then resuspended in 0.56% KCl solution, fixed using methanol/glacial acetic acid at a ratio of 3:1 v/v, stained with Diff Quik (Sysmex Corporation), mounted with Neo‐Mount Anhydrous Mounting Medium, and then analyzed for metaphase plate. Twenty‐five microgram per milliliter of cyclophosphamide (Sigma‐Aldrich) with metabolic activation and 2.5 μg/ml of mytomycin C without metabolic activation were used as positive controls. At least 100 well‐spread metaphases per dish were analyzed and a positive result is considered if there was a dose‐dependent increase and if the frequency of aberrant cells was >3%. Types of structural chromosomal aberrations include chromosome gap (G), chromosome break (B), chromosome dicentric (D), chromosome ring (R), chromatid gap (g), chromatid break (b), and chromatid exchange (e).

### 2.7 Animal husbandry

All animal experiments were carried out after receiving approval from the Institutional Animal Care and Use Committee and conducted in compliance with Good Laboratory Practice for nonclinical laboratory studies (FDA, 2011). The animals (males weighing 34 ± 3 g and females weighting 25 ± 2 g) were obtained from BioLASCO Taiwan Co., housed randomly in polypropylene cages (*n* = 2), and allowed 1 week to adapt to their environment prior to testing. The mice were maintained in a temperature‐controlled room of 23 ± 2°C under a 12 hr light–dark cycle with ad libitum access to standard rodent diet (1324N; Altromin) and purified water.

### 2.8 Acute oral toxicity assay

The acute toxicity test was performed as per OECD Guideline for the testing of chemicals #423: Acute oral toxicity (OECD, 2002). A dose of 12 g kg^−1^ day^−1^ was selected as the toxicological limited dose as the preliminary study showed that acute oral LD_50_ of hispidin‐enriched *S. sanghuang* mycelia did not induce any toxic effect. Twenty healthy ICR mice were assigned to two groups (five males and five females per group), and treated with a single dose of 0 and 12 g kg^−1^ day^−1^ of hispidin‐enriched *S. sanghuang* mycelia, respectively. Animals were observed individually for signs of toxicity, mortality, morbidity, and body weight changes for 7 days. At the end of the experiment, all overnight fasted mice were sacrificed under carbon dioxide anesthesia, and the blood and organs were collected for hematology, clinical biochemistry, and histopathological examination. This study was approved by the Institutional Animal Care and Use Committee (No. 103‐08).

### 2.9 Bone marrow micronucleus test

The bone marrow micronucleus test was performed in compliance with OECD Guideline for the testing of chemicals #474: Mammalian Erythrocyte Micronucleus Test (OECD, 1997c). Based on the acute toxicity test results, 5 g/kg of *S. sanghuang* mycelia was selected as the highest dose for the bone marrow micronucleus test. Low, mid, and high dose groups of five male ICR mice were administered hispidin‐enriched *S. sanghuang* mycelia orally by gavage at doses of 1.25, 2.50, and 5 g/kg body weight, respectively. Negative controls received sterilized water while positive controls were given an intraperitoneal injection of 50 mg/kg cyclophosphamide. Peripheral blood was collected at 48 and 72 hr after administration of the treatments, and 3–4 μl drops were smeared on a glass microscope slide stained with acridine orange. Examination of the slides was conducted with fluorescence microscopy. A total of 2,000 cells per mouse were examined, and micronucleated cells were counted.

### 2.10 Statistical analysis

The Ames test, in vitro chromosomal aberrations, and bone marrow micronucleus test were expressed as mean ± standard deviation (*SD*), and the acute toxicity data were presented as mean ± standard error of the mean (*SEM*). All data were analyzed using one‐way analysis of variance (ANOVA). Significant differences between the control and experimental groups were determined by the Dunnett multiple comparison tests, and a *p*‐value < .05 was considered statistically significant.

## 3 RESULTS

### 3.1 Genotypic identification of the *S. sanghuang* strain

**Figure 1:**
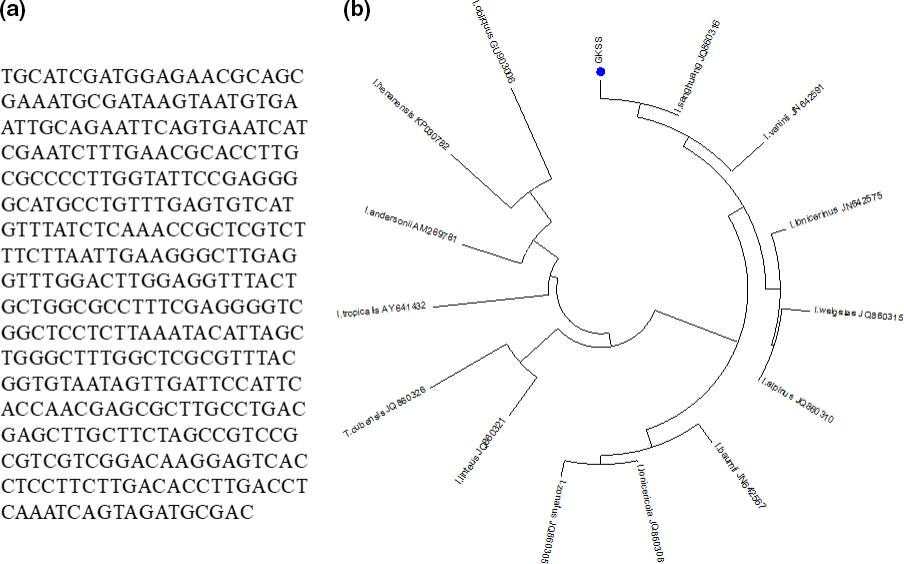
(a) Nucleotide sequence and (b) phylogenetic tree for *Sanghuangporus sanghuang* (GKSS) strain

The nrDNA ITS region (Figure 1a) of the strain used in this study (GKSS) was amplified using ITS1 and ITS4 primers and analyzed using the phylogenetic method to ascertain the identity of the strain. The BLAST searches of the NCBI nucleotide database indicated that the ITS sequence of the GKSS strain exhibited a 99.2% similarity with *P. linteus* (AF200227.1) and *S. sanghuang* (MG062789.1) isolates. In the phylogenetic analyses (Figure 1b), the strain of this study formed a clade with *S. sanghuang* isolate (JQ860316) with 100% bootstrap support. Based on these results, GKSS was confirmed to be a *S. sanghuang* strain.

### 3.2 Identification of hispidin from *S. sanghuang* mycelia extract

**Figure 2:**
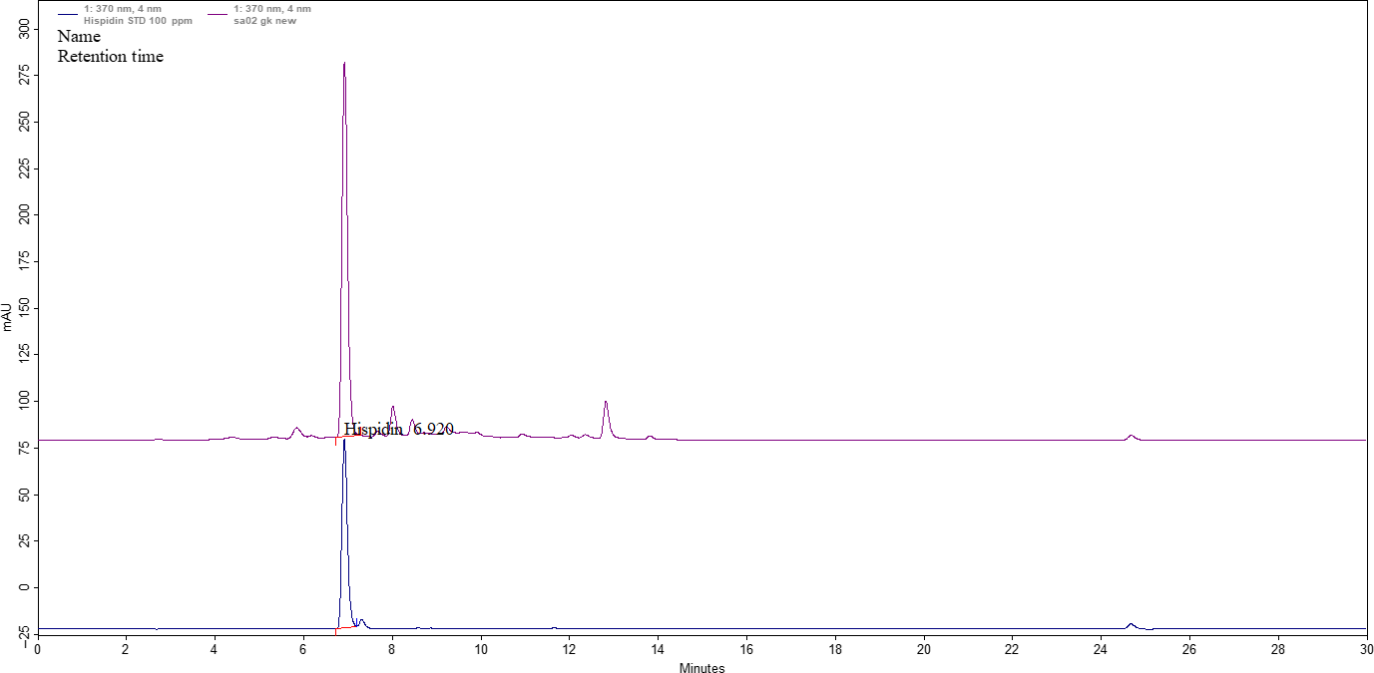
HPLC chromatogram of the standard (bottom) and hispidin (top) extracted from *Sanghuangporus sanghuang* mycelia

In Figure 2, the retention time for hispidin and its standard was detected at 6.920 min. No significant interference from other metabolites was observed in the fermentation extracts at the retention time of hispidin or the standard. Therefore, this HPLC chromatographic condition provided adequate specificity to estimate the production of hispidin present in fermentation extracts.

### 3.3 Selection of favorable culture media using flasks

**Figure 3:**
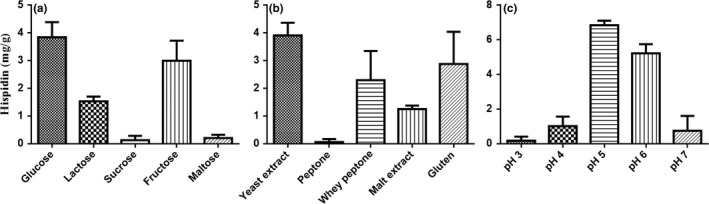
Effects of (a) carbon sources, (b) nitrogen sources, and (c) pH on the hispidin production of *Sanghuangporus sanghuang* mycelia

Fifteen different culture media (250 ml in volume) consisting of five carbon sources, five nitrogen sources, and five initial pH conditions were utilized to determine the optimal hispidin production of *S. sanghuang* mycelia. First, each carbon source instead of glucose (as control) was added to the basal medium consist of same concentration of yeast extract and MgSO_4_. Among the five carbon sources utilized, the medium with glucose was shown to be exceedingly favorable in promoting hispidin production of *S. sanghuang* mycelia (Figure 3a). Next, the culture was carried out in flasks containing the basal medium with various nitrogen sources and same concentration of glucose and MgSO_4_. The nitrogen source that most effectively promoted hispidin production of *S. sanghuang* mycelia was yeast extract (Figure 3b). Finally, the influence initial pH on hispidin production in a medium containing glucose, yeast extract, and MgSO_4_ was investigated in flasks. The most favorable hispidin production of *S. sanghuang* mycelia was noted at a pH of 5 (Figure 3c). These optimized media were subsequently carried out in a large‐scale production system (20‐ton bioreactor) for further studies, which resulted in a yield of 3 mg/g hispidin (data not shown).

### 3.4 3 mg/g hispidin‐enriched *S. sanghuang* mycelia treatment caused no genotoxicity in vitro

The mutagenicity of the 3 mg/g hispidin‐enriched *S. sanghuang* mycelia was investigated in *S. typhimurium* test strains TA98, TA100, TA102, TA1535, and TA1537 in the presence and absence of metabolic activation. All five different concentrations of 3 mg/g hispidin‐enriched *S. sanghuang* mycelia did not increase the number of revertants of TA98, TA100, TA102, TA1535, and TA1537 when compared to negative controls (*p* > .05; Table 1), suggesting that 3 mg/g hispidin‐enriched *S. sanghuang* mycelia were not mutagenic in these *S. typhimurium* strains in both the presence and absence of S9 mix. Additionally, the effects of 3 mg/g hispidin‐enriched *S. sanghuang* mycelia at the sublethal concentrations (at least 50% reduction in degree of confluency) on chromosome aberrations were evaluated in CHO‐K1 cells in the presence and absence of S9 mix (Table 2). Results showed that neither short‐term (3 hr) nor long‐term (20 hr) treatment induced higher frequency of aberrations when compared to negative controls (*p* > .05), suggesting that exposure to the 3 mg/g hispidin‐enriched *S. sanghuang* mycelia did not induce chromosome aberrations under the conditions tested.

**Table 1:** Number of revertant colonies after 3 mg/g hispidin‐enriched *Sanghuangporus sanghuang* mycelia exposure in the absence and presence of S9 mix

Dose (µl/plate)	No. of histidine revertants/plate				
	TA98	TA100	TA102	TA1535	TA1537
+S9					
Spontaneous reversion rate	42 ± 2	128 ± 15	251 ± 9	14 ± 2	15 ± 3
*S. sanghuang* mycelia 0.3125	38 ± 3	137 ± 6	264 ± 15	14 ± 1	15 ± 3
*S. sanghuang* mycelia 0.625	43 ± 3	128 ± 19	272 ± 23	16 ± 3	15 ± 4
*S. sanghuang* mycelia 1.25	43 ± 6	135 ± 3	264 ± 21	18 ± 4	16 ± 2
*S. sanghuang* mycelia 2.5	45 ± 4	136 ± 5	288 ± 22	17 ± 2	15 ± 3
*S. sanghuang* mycelia 5	44 ± 2	137 ± 10	260 ± 25	17 ± 2	17 ± 2
Positive controls^a^	334 ± 12^b^	449 ± 28^b^	598 ± 19^b^	155 ± 10^b^	397 ± 13^b^
−S9					
Spontaneous reversion rate	30 ± 2	137 ± 8	220 ± 24	20 ± 5	10 ± 2
*S. sanghuang* mycelia 0.3125	23 ± 2	131 ± 4	204 ± 12	16 ± 2	12 ± 1
*S. sanghuang* mycelia 0.625	28 ± 5	139 ± 6	209 ± 6	15 ± 2	12 ± 1
*S. sanghuang* mycelia 1.25	29 ± 5	138 ± 5	202 ± 13	17 ± 3	12 ± 2
*S. sanghuang* mycelia 2.5	31 ± 3	131 ± 3	205 ± 5	18 ± 3	14 ± 2
*S. sanghuang* mycelia 5	30 ± 2	134 ± 7	202 ± 10	20 ± 2	13 ± 2
Positive controls^c^	316 ± 20^b^	970 ± 28^b^	929 ± 34^b^	779 ± 32^b^	524 ± 9^b^

Note

Results are expressed as mean ± *SD* of triplicates.

a TA98, TA102: Benzo[a]pyrene, 4 μg/plate; TA100: 2‐aminofluorene, 4 μg/plate; TA1535; TA1537: 2‐aminoanthracene, 4 μg/plate.

b Twofold increase or more in revertant numbers over the negative control.

c TA98: 4‐nitroquinoline‐N‐oxide, 0.5 μg/plate; TA100 and TA1535: sodium azide, 0.4 μg/plate; TA102: Mitomycin C, 0.5 μg/plate; TA1537: 9‐aminoacridine, 50 μg/plate.

**Table 2:** Effects of 3 mg/g hispidin‐enriched *Sanghuangporus sanghuang* mycelia in chromosomal aberration in CHO‐K1 cells

Dose (%)	S9‐Mixure	G	B	D	R	g	b	Int	Itr	Other	AF^a^
Short‐term treatment (3 hr)											
Negative control^b^	+	0	0	0	0	0	0	0	0	0	0/200
Positive control^c^	+	0	3	13	0	0	5	4	1	0	26/200*
*S. sanghuang* mycelia 1.25	+	0	1	0	0	0	0	0	0	0	1/200
*S. sanghuang* mycelia 0.625	+	0	0	0	1	0	1	0	0	0	2/200
*S. sanghuang* mycelia 0.3125	+	0	0	0	0	0	0	0	0	0	0/200
Negative control^b^	−	0	0	0	0	0	0	0	0	0	0/200
Positive control^d^	−	0	3	2	1	0	6	23	4	0	39/200*
*S. sanghuang* mycelia 5	−	0	0	0	0	0	0	0	0	0	0/200
*S. sanghuang* mycelia 2.5	−	0	0	0	0	0	0	0	0	0	0/200
*S. sanghuang* mycelia 1.25	−	0	0	0	0	0	0	0	0	0	0/200
Long‐term treatment (20 hr)											
Negative control^b^	−	0	0	0	0	0	0	0	0	0	0/200
Positive control^d^	−	0	1	3	0	0	11	10	3	0	28/200*
*S. sanghuang* mycelia 5	−	0	0	0	0	0	0	0	0	0	0/200
*S. sanghuang* mycelia 2.5	−	0	0	0	0	0	0	0	0	0	0/200
*S. sanghuang* mycelia 1.25	−	0	0	0	0	0	1	0	0	0	1/200

Note

Chromosome gaps were recorded separately but not included in aberrant cells. b, chromatid break; B, chromosome break; D, dicentric; g, chromatid gap; G, chromosome gap; Int, interchange; Itr, intrachange; R, ring.

a Aberration frequency: Number of cells with chromosome aberration in 200 metaphase cells (*n*/200).

b Ham's F‐12 Culture medium with 10% fetal bovine serum.

c 25 μg/ml cyclophosphamide.

d 2 μg/ml mitomycin C.

* Statistically significant (*p* < .05) when compared to the control group.

### 3.5 No deaths or abnormalities observed in the single‐dose acute toxicity study

**Figure 4:**
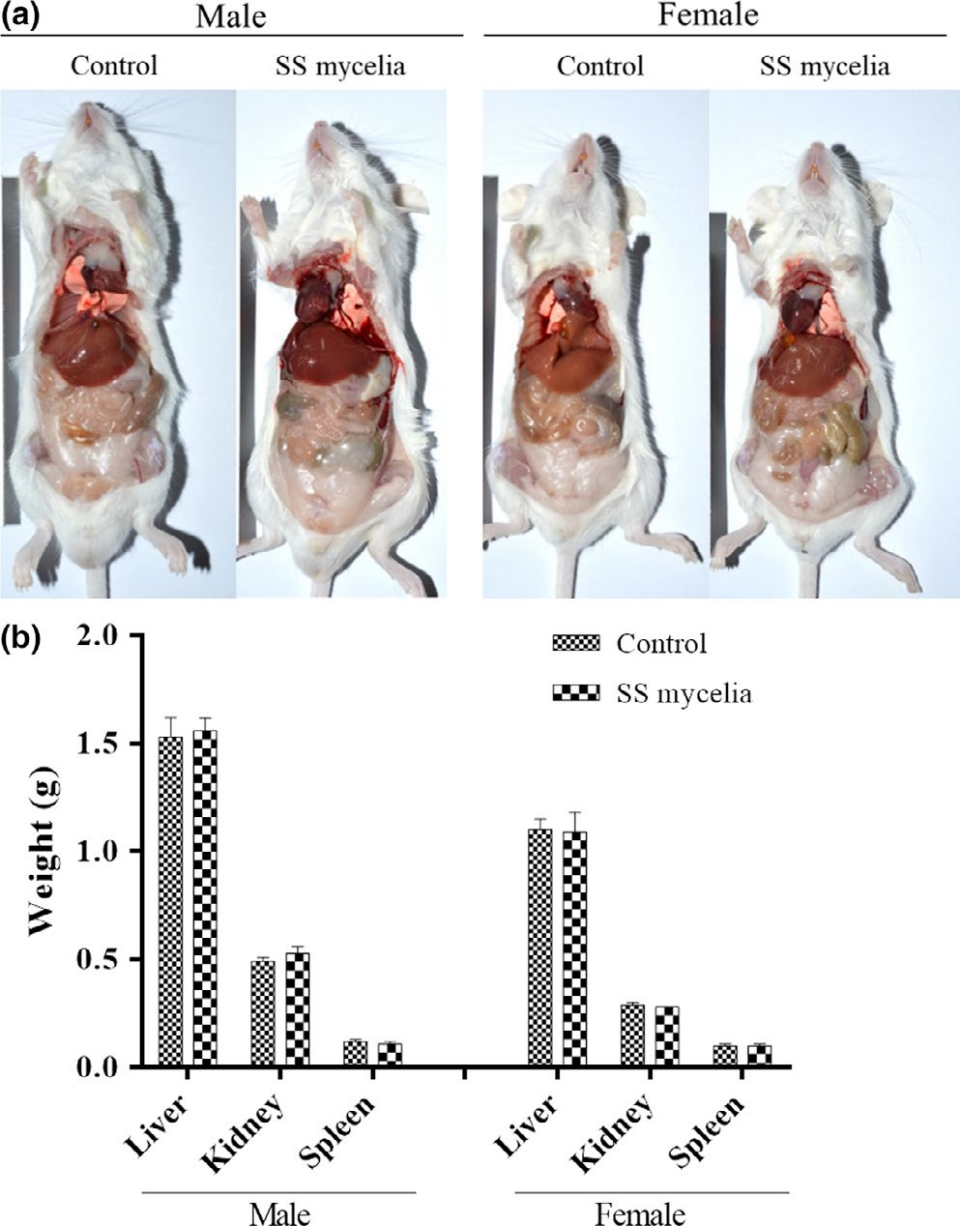
(a) Gross pathology and (b) organ weights in male and female mice treated with a single oral gavage dose of 3 mg/g hispidin‐enriched *Sanghuangporus sanghuang* (SS) mycelia

In the acute toxicity study, administration of 3 mg/g hispidin‐enriched *S. sanghuang* mycelia at a single dose of 12 g kg^−1^ day^−1^ did not cause mortality during the observational period of 7 days. No significant changes in weight, ALT, and AST (*p* > .05; Table 3) were detected in the males and female mice. Although there is a statistically significant decrease in BUN level in male mice exposed to 3 mg/g hispidin‐enriched *S. sanghuang* mycelia, this value fell well within the normal reference range (Serfilippi, Pallman, & Russell, 2003). Concurrently, no noticeable significant difference in the gross pathology (Figure 4a), organ weights (Figure 4b), and histopathology (data not shown) of treated mice were shown after 7 days of treatments compared with the control groups, indicating that the mean lethal dose (LD_50_) of this mycelia is >12 g/kg.

**Table 3:** Effects of 3 mg/g hispidin‐enriched *Sanghuangporus sanghuang* mycelia on clinical pathology parameters in single‐dose acute toxicity study

Parameters	Control	PL mycelia
Male		
Day 0 weight (g)	35.2 ± 0.6	36.3 ± 0.6
Day 7 weight (g)	37.8 ± 0.9	38.3 ± 0.9
ALT (U/L)	51.8 ± 2.5	58.3 ± 5.5
AST (U/L)	105.6 ± 6.2	108.7 ± 7.5
BUN (mg/dl)	29 ± 0.6	20.2 ± 1.1*
Female		
Day 0 weight (g)	27 ± 0.4	26.6 ± 0.6
Day 7 weight (g)	27.5 ± 0.4	26.7 ± 0.4
ALT (U/L)	56.6 ± 3.1	52.5 ± 1.6
AST (U/L)	108.6 ± 7.7	87.7 ± 3.5
BUN (mg/dl)	20.8 ± 1.7	17.5 ± 0.8

Note

Results are expressed as mean ± *SD* (*n* = 5) and analyzed by one‐way ANOVA and Duncan's multiple range test.

Abbreviations: ALT, alanine aminotransferase; AST, aspartate aminotransferase; BUN, blood urea nitrogen.

* Significantly different from the control at *p* < .05.

### 3.6 Chromosome damages not found using micronucleus tests in mice

Based on the acute toxicity test results, animals were grouped to receive treatments of negative control (distilled water), positive control (0.05 g/kg cyclophosphamide), and 3 mg/g hispidin‐enriched *S. sanghuang* mycelia in dose levels of 1.25, 2.5, and 5 g/kg. After 48‐ and 72‐hr post‐treatment, the frequencies of reticulocytes in red blood cells and micronucleated reticulocytes in reticulocytes were calculated. All dosages did not change the abundance of reticulocytes and did not increase the rate of micronucleated reticulocytes significantly (*p* > .05; Table 4) at both 48‐ and 72‐hr post‐treatment, suggesting that 3 mg/g hispidin‐enriched *S. sanghuang* mycelia ingestion does not lead to chromosomal damages during cell cycle processes.

**Table 4:** Effects of 3 mg/g hispidin‐enriched *Sanghuangporus sanghuang* mycelia on mouse bone marrow erythrocytes

Dose (g/kg)	RETs/1,000 RBCs	Mn‐RETs/1,000 RETs
48 hr		
Negative control	49.6 ± 1.5	0.4 ± 0.5
Positive control^a^	21.2 ± 1.6*	19.6 ± 1.5*
1.25	49.8 ± 1.5	0.6 ± 0.5
2.5	50.0 ± 1.6	0.4 ± 0.5
5	49.4 ± 1.7	0.6 ± 0.5
72 hr		
Negative control	50.0 ± 1.2	0.6 ± 0.5
Positive control^a^	20.6 ± 1.5*	20.2 ± 1.6*
1.25	50.0 ± 1.9	0.2 ± 0.4
2.5	50.2 ± 1.3	0.8 ± 0.8
5	50.2 ± 1.8	0.4 ± 0.5

Note

Results are expressed as mean ± *SD* (*n* = 5) and analyzed by one‐way ANOVA and Duncan's multiple range test.

Abbreviations: Mn‐RETs, micronucleated reticulocytes; RBCs, red blood cells; RETs, reticulocytes.

a 100 mg/kg cyclophosphamide.

* Significantly different from the control at *p* < .05.

## 4 DISCUSSION

Fungi are known to produce a wealth of bioactive secondary metabolites and have a wide range of applications in agriculture, industrial, pharmaceuticals, and biomedical sectors (Fouda, Hassan, Eid, & Ewais, 2015; Sheng et al., 2019). To date, over 750 new metabolites from fungi have been described between 1993 and 2013, which confirms the tremendous potential of the fungal secondary metabolome (Keller, 2019). While these compounds display an array of biologic activities that contribute to the survival of the producing organism, they may also be regarded as promising leads for new drug development (Dias, Urban, & Roessner, 2012; Garza et al., 2019). Some notable examples with pharmaceutical significance and industrial impact include cephalosporins, lovastatin, cyclosporine, and myriocin (Alberti, Foster, & Bailey, 2017). In fact, to guarantee that these drugs are reproducible, it is critical to ensure that the taxonomic identification of the fungi is correct. Accurate species identification not only can unlock important scientific information of the species but also its possible biosynthetic pathways for secondary metabolites. More broadly, this in turn can provide insight into developing better screening programs of natural products in drug discovery.

*Phellinus linteus* is an economically valuable traditional medicinal mushroom with various medicinal functions (Chen et al., 2016). However, the identity of this species has been called into question lately due to its multiple taxon concepts and the inadequacy of morphological distinctions within closely allied species (Han et al., 2016). As the identification of fungal taxonomy has been based on morphology characters such as spore‐producing structures, it could often be misleading due to several factors, such as hybridization (Hughes et al., 2013), cryptic speciation (Lucking et al., 2014), and convergent evolution (Brun & Silar, 2010). Hence, DNA sequence‐based identification methods have emerged to distinguish each unique species among other phyla within the megadiverse fungi kingdom (Stajich et al., 2009).

Lately, a previous work based on molecular phylogenetic analysis showed that *Phellinus* is not a monophyletic group and subsequently grouped *P. baumii*, *P. linteus*, *P. pachypholeus*, *P. tropicalis*, *P. vaninii*, and *P. weirianus* into the same genus *Inonotus* (Larsson et al., 2006). Nevertheless, as all examined specimens named *I. linteus* or *I. baumii* were not collected on the tree *Morus* mentioned in early Chinese folklore, Wu et al. (2012) discovered that the true sanghuang that grows solely on *Morus* was a previously undescribed species and hence designated it as a new species, *I. sanghuang*. Recently, a comprehensive study using morphological and phylogenetic data of global samples revealed that *Inonotus* not only is a polyphyletic genus but also comprises at least three clades (Zhou et al., 2016). Among these clades, the true sanghuang (*I. sanghuang*) belongs to a member of *Sanghuangporus* (Zhou et al., 2016). Since the sanghuang species concept has been clearly elucidated, it has become necessary to clarify the taxonomic position of the strain used in this study (GKSS). Based on the nucleotide sequences and the phylogenetic analysis of the nrDNA ITS region, the GKSS strain is confirmed as *S. sanghuang*.

While the confirmation of identity is a critical starting point in the overall process for the safety evaluation of fungal raw materials, the secondary metabolite profile of fungi should also be assessed as they are crucial players in the fungal development (Tannous et al., 2016). Considering that many fungi metabolites are of medical, industrial, and agricultural importance (Calvo, Wilson, Bok, & Keller, 2002), optimization of the growing environment can significantly increase the yield of a compound of interest in the industrial setting. The yellow‐brown styrylpyrone pigment hispidin has garnered significant attention due to its antioxidant (Park et al., 2004), anti‐obesity (Tu & Tawata, 2014), anticancer (Lim et al., 2014), antiviral (Awadh Ali et al., 2003), anti‐allergic (Tamrakar et al., 2018), antibacterial, and anti‐inflammatory effects (Shao et al., 2015). In this study, the optimal medium for hispidin production in flasks was observed at the composition of 1% glucose, 0.3% yeast extract, and 0.05% MgSO_4_, adjusted to pH 5. Using the optimized medium, hispidin production was achieved at 3 mg/g after 10 days of fermentation in a 20‐ton bioreactor. These results showed that a medium with a carbon to nitrogen (C/N) ratio of 3 and a pH value of 5 may be important parameters in promoting the biosynthesis of hispidin in *S. sanghuang* mycelia. More importantly, given that hispidin production in a 20‐ton bioreactor has a certain level of agreement with laboratory‐scale results, it suggests that the potential for implementation and industrial exploitation of target molecules on a commercial scale are possible.

In anticipation of the commercialization of hispidin‐enriched *S. sanghuang* in a product, a series of safety evaluations must be initiated. Ames test (Maron & Ames, 1983), in vitro chromosome aberration test (OECD, 1997b), acute oral toxicity test (OECD, 2002), and bone marrow micronucleus test (OECD, 1997c) are the standard tests to detect toxicological properties of drug candidates. In this study, 3 mg/g hispidin‐enriched *S. sanghuang* mycelia showed no mutagenic activity in the bacterial reverse mutation test and caused no significant increases in the number of structural aberrations in CHO‐K1 cells. Moreover, the results of acute (7 days) oral toxicity studies of 3 mg/g hispidin‐enriched *S. sanghuang* mycelia in mice confirmed in part the safety of this ingredient for oral consumption (LD_50_ > 12 g/kg b.w). Furthermore, oral administration of 3 mg/g hispidin‐enriched *S. sanghuang* mycelia to ICR mice at a dose of 5 g/kg did not increase the number of reticulocytes in red blood cells and micronucleated reticulocytes in reticulocytes. These results demonstrated the safe profile of 3 mg/g hispidin‐enriched *S. sanghuang* mycelia for human consumption, inferred from the absence of any remarkable adverse effects in mice.

The results of the present study concluded that glucose and yeast extract at an initial pH of 5 are the optimal conditions for the production of hispidin by *S. sanghuang* mycelia. Moreover, the absence of any remarkable adverse effects in the Ames test, in vitro chromosome aberration test, acute oral toxicity test, and bone marrow micronucleus test demonstrated that 3 mg/g hispidin‐enriched *S. sanghuang* mycelia is safe for human consumption. Nevertheless, future studies on the mechanisms of 3 mg/g hispidin‐enriched *S. sanghuang* mycelia within pharmacological parameters should be useful to further delineate its role in the prevention and treatment of diseases.

## CONFLICT OF INTEREST

The authors declare that they do not have any conflict of interest.

## ETHICAL APPROVAL

The study was carried out after the study's protocols, and procedures were ethically reviewed and approved from the Institutional Animal Care and Use Committee (No. 103‐08). All animal experiments received care in compliance with Good Laboratory Practice for nonclinical laboratory studies.
